# Vascular Endothelial Growth Factor Receptor-2 Polymorphisms Have Protective Effect against the Development of Tendinopathy in Volleyball Athletes

**DOI:** 10.1371/journal.pone.0167717

**Published:** 2016-12-08

**Authors:** José Inácio Salles, Maria Eugenia Leite Duarte, João Matheus Guimarães, Lucas Rafael Lopes, Jessica Vilarinho Cardoso, Diego Pinheiro Aguiar, João Olyntho Machado Neto, Daniel Escorsim Machado, Jamila Alessandra Perini

**Affiliations:** 1 Divisão de Pesquisa, Instituto Nacional de Traumatologia e Ortopedia, Rio de Janeiro, Brasil; 2 Federation International de Volleyball (FIVB)—Coach Commission, Rio de Janeiro, Brasil; 3 Laboratório de Pesquisa de Ciências Farmacêuticas, Unidade de Farmácia, Centro Universitário Estadual da Zona Oeste, Rio de Janeiro, Brasil; 4 Programa de Pós-Graduação em Saúde Pública e Meio Ambiente, Escola Nacional de Saúde Pública, Fundação Osvaldo Cruz, Rio de Janeiro, Brasil; 5 Brazilian Volleyball Federation, Rio de Janeiro, Brasil; Universidad Europea de Madrid, SPAIN

## Abstract

The aim of the study was to investigate whether genetic variants in *VEGF* and *KDR* genes can be correlated with susceptibility of tendinopathy in volleyball athletes. This study was conducted at the Brazilian Volleyball Federation, and comprised 179 volleyball athletes: 88 had a confirmed diagnosis of tendinopathy (cases), whereas 91 had no evidence of the disease (controls). The *VEGF* (-*2578C>A*, *-460T>C* and *+936C>T*) and *KDR* (-*604C>T*, *1192G>A* and *1719T>A*) polymorphisms were determined by TaqMan real-time polymerase chain reaction. The odds ratio (OR) with their 95% confidence intervals (CI) were calculated using an unconditional logistic regression model. The evaluation of demographic and clinical characteristics revealed the athlete age (*P* < 0.001), years of practice in volleyball (*P* < 0.001) and presence of pain (*P* = 0.001) were risk factors for tendinopathy. *KDR 1192 GA* and *GA* + *AA* genotypes were associated with lower risk of tendinopathy (OR: 0.41, 95% CI: 0.19–0.88 and OR: 0.47, 95% CI: 0.23–0.98, respectively). The *KDR* (-*604C>T*, *1192G>A* and *1719T>A*) haplotypes *CGA* and *CAT* were associated with decreased tendinopathy risk (OR: 0.46, 95% CI: 0.21–0.99 and OR: 0.23, 95% CI: 0.07–0.76, respectively). With regards to pain, traumatic lesion and away from training due to injury, *VEGF* and *KDR* polymorphisms were not associated with clinical symptoms complaints. The present results provide evidence that the *KDR* polymorphisms were associated with development of tendinopathy, and can contribute to identify new therapeutic targets or personalized training programs to avoid tendinopathy development in athletes.

## Introduction

Tendinopathy is a common injury caused by overuse, particularly among athletes or individuals engaging in repetitive activities, and corresponds to around 45% of all musculoskeletal disease [1]. The major concerns of chronic tendon injuries for athletes are: their need to remain away from training due to injury, their quality of life and professional performance, and health care expenditure [2]. The etiology of tendinopathy is regarded as being unclear, despite genetic [3–7] and extrinsic factors that may contribute to the phenotype [1]. Many studies have shown an increase in angiogenesis in degenerative tendons [8, 9]. The Vascular Endothelial Growth Factor (VEGF) is the most potent angiogenic factors in both physiologic and pathologic angiogenesis. VEGF signaling is mediated by two tyrosine kinase receptors: VEGF receptor-1 (VEGFR-1) and VEGF receptor-2 (VEGFR-2), the latter also being called kinase insert domain containing receptor (KDR), although VEGF-KDR interaction is the most important one for angiogenesis and multiple signaling cascades are activated by VEGFR-2 [10]. Interestingly, VEGF expression is detected in tendons of patients with degenerative tendinopathies but not in normal tendons [11, 12]. Furthermore, VEGF over expression is essential in an attempt to repair injuries. Unfortunately, the accumulation of VEGF results in a degenerative process. VEGF stimulates the invasion of blood vessels therefore weakening the structure of the normal tendon, which leads to a decreased mechanical strain and subsequently to spontaneous rupture [13].

The expression of *VEGF* and *KDR* genes are highly modulated, and single nucleotide polymorphisms (SNP) are present inside the promoter region (*VEGF* rs699947, rs833061 and *KDR* rs2071559), and in the 3' (*VEGF* rs3025039) untranslated region (UTR), next to many potential regulatory elements [14, 15]. In addition, two exonic SNPs (*KDR* rs2305948 and rs1870377), which result in nonsynonymous amino acid changes, have been reported to influence the efficiency of VEGF binding to KDR [14].

Recently, there has been growing interest in investigating the role of VEGF and its receptor in tendinopathy [16–18]; as both genes are polymorphic, it may affect the inheritable susceptibility to development of tendinopathy. Therefore, the present study aimed to explore *VEGF* and *KDR* SNPs as potential biomarkers for tendinopathy susceptibility among Brazilian volleyball athletes. Understanding the molecular mediators that lead to tendinopathy and subsequently could contribute to an increased risk or severity of the disease, is essential for providing additional support for the personalized treatment to avoid tendinopathy in athletes.

## Methods

### Participants

The case-control study was approved by the Human Research Ethics Committee of the National Institute of Traumatology and Orthopedics, Brazil (Protocol number 17373613.8.0000.5273/2013). All participating athletes (n = 179) provided written informed consent and answered a questionnaire about their demographics, medical history, personal tendon injury history, painful symptoms and sports activities. Data were obtained by in-person interviews carried out from January through July 2014. Inclusion criteria were volleyball players, male or female, belong to the Brazilian Volleyball Federation. The athletes were divided into two groups (cases and controls) regarding the presence or absence of previously diagnosed tendinopathy at any site. Eighty-eight athletes with tendinopathy in patellar (n = 53, 60.2%), shoulder (n = 21, 23.9%) and Achilles (n = 14, 15.9%) confirmed with magnetic resonance image examination of the affected tendon were enrolled as cases. According to our previous report [7], the clinical diagnostic criteria for chronic tendinopathy were (i) progressive pain related to training in the last 6 months and during clinical examination; and at least one of the following criteria: (ii) palpable nodular thickening over the tendon; (iii) tenderness on tendon palpation; (iv) history of swelling over the tendon area. Controls (n = 91) were athletes without history of tendinopathy in any joint and who reported no previous diagnosis of tendinopathy.

### *VEGF* and *KDR* genotyping

Genomic DNA was obtained from saliva samples as previously described [7]. A validated TaqMan assay (VIC- and FAM-labeled) for detection of each *VEGF -2578C>A* (rs699947), *-460T>C* (rs833061), +*936C>T* (rs3025039) and *KDR -604C>T* (rs2071559), *1192G>A* (rs2305948) and *1719T>A* (rs1870377) SNPs was purchased from Applied Biosystems. Table 1 summarizes the sets of probes and primers used for each *KDR* SNPs analysis, because *VEGF* SNP Genotyping Assays has been already previously described [19]. The allele-detection processes were performed on a 7500 Real-Time System (Applied Biosystems, Foster City, CA, USA) to determine the allelic discrimination.

**Table 1:** Characterization of *KDR* polymorphisms, probes and primers sequences for genotyping by TaqMan real time PCR.

Identified SNP	TaqMan assays	Region	Probe [SNP]	Primer
rs2071559	C__15869271_10	PR	GGTATGGGTTTGTCACTGAGACAGC[A/T]TGGCTATAAGAAAGAGATAACAGCG	Forward: 5'-CCTCCTGTATCCTGAATGAATCT-3'
				Reverse: 5'-GCCTCACATATTATTGTACCATCC-3'
rs2305948	C__22271999_20	Exon 7	AATATTTTGGGAAATAGCGGGAATG[C/T]TGGCGAACTGGGCAAGTGCGTTTTC	Forward: 5'-CAAACTTTCACTAGGGCTCTTCGT-3'
				Reverse:5'-AGCCACAAGGGAGAAGCGGATA-3'
rs1870377	C__11895315_20	Exon 11	TACAATCCTTGGTCACTCCGGGTTA[C/T]ACCATCTATAGTTAAGGTGCTCAAA	Forward: 5'-TGAGGTTAAAAGTTCTGGTGTCCCTGTT-3'
				Reverse: 5'-AATGTACAATCCTTGGTCACTCCGGGGTA-3'

PR is Promoter Region.

### Statistical analysis

The sample size was calculated using Epi Info 7, version 7.1.3. (http://wwwn.cdc.gov/epiinfo/html/downloads.htm) to detect a difference between case and control groups, assuming an odds ratio of 1.2 with a power of 0.8 and 5% type I error.

Comparisons of age and years of practice in volleyball in both groups were performed using the Stundent’s t test, and data were presented as mean ± standard deviation (SD). The nominal data, such as age, years of practice in volleyball, gender, declared preference, volleyball function, traumatic lesion, pain, as well as the away from training due to injury, were expressed as percentages and evaluated by Chi-Square Test or Fisher’s exact test, when applicable.

Deviations from Hardy–Weinberg equilibrium (HWE) were assessed by the goodness-of-fit χ2 test. *VEGF* and *KDR* allele frequency and genotype distribution were derived by gene counting. Allele and genotype frequencies between the two groups were compared using the χ2 test or, when appropriate, the Fisher’s exact test. The haplotype patterns and linkage disequilibrium coefficients (D’ is degree of imbalance in module and R^2^ is degree of correlation) were inferred using Haploview, as previously described [19].

The risk associations for tendinopathy were estimated by the odds ratio (OR) with 95% confidence interval (95% CI). Confounding factors that could potentially influence the risk for tendinopathy (P = 0.20) were taken into account in multivariate logistic regression models. All statistical analyses were conducted using Statistical Package for Social Sciences (SPSS Inc., Chicago, IL, USA) for Windows, version 15.0 and a *P* value less than 0.05 was considered statistically significant.

## Results

The demographic and clinical variables of tendinopathy cases and controls are presented in Table 2 (S1 Table). In summary, tendinopathy cases were significantly older than controls (23.0 ± 4.1 versus 20.2 ± 5.1, *P* < 0.001), with higher years of practice in volleyball (9.0 ± 3.7 versus 7.4 ± 4.6, *P* < 0.01), higher prevalence of pain in any joint and more away from training due to injury.

**Table 2:** Demographic and clinical characteristics of the volleyball athletes.

Variables	Controls (n = 91)	Tendinopathy (n = 88)	*P*-value^a^
Age (years)	N (%)		
Sub 18	46 (50.5)	14 (15.9)	< 0.001
Sub 23	29 (31.9)	33 (37.5)	
Adult	16 (17.6)	41 (46.6)	
Years of practice in volleyball			
0–5	40 (44.0)	17 (19.3)	< 0.001
6–10	32 (35.2)	39 (44.3)	
11–15	12 (13.2)	31 (35.2)	
> 15	7 (7.7)	1 (1.1)	
Gender of the athletes			
Female	52 (57.1)	29 (33.0)	0.001
Male	39 (42.9)	59 (67.0)	
Declared preference			
Right	88 (96.7)	86 (97.7)	0.68
Left	3 (3.3)	3 (2.3)	
Function			
Spiker	69 (75.8)	71 (80.7)	0.73
Setter	16 (17.6)	12 (13.6)	
Libero	6 (6.6)	5 (5.7)	
Pain in any joint			
No	31 (34.1)	12 (13.6)	0.001
Yes	60 (65.9)	76 (86.4)	
Traumatic lesion			
No	60 (65.9)	58 (65.9)	0.99
Yes	31 (34.1)	30 (34.1)	
Away from training due to injury			
No	64 (70.3)	49 (55.7)	0.04
Yes	27 (29.7)	39 (44.3)	

^a^Chi-Square Test or Fisher’s exact test.

**Fig 1:**
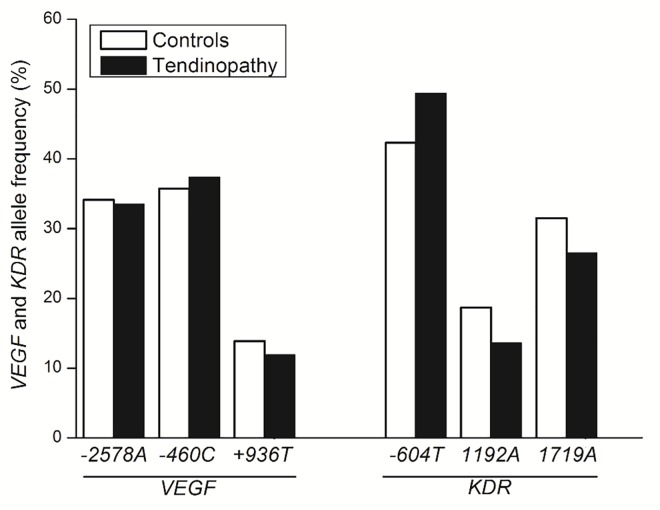
Allelic frequency of *VEGF* and *KDR* polymorphisms in tendinopathy cases and controls.

**Fig 2:**
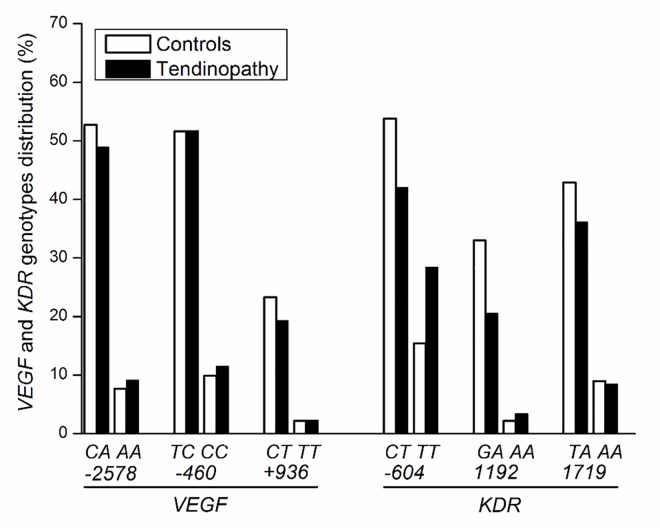
Genotypic distribution of *VEGF* and *KDR* polymorphisms in volleyball athletes.

The *VEGF -2578C>A*, *-460T>C*, *+936C>T* and *KDR -604C>T*, *1192G>A* and *1719T>A* SNPs were in HWE in the overall athletes study population (S1 Table) and in each group (tendinopathy cases and controls). The allelic and genotypic frequencies of all *VEGF* and *KDR* SNPs in the study population are shown in Figs 1 and 2, respectively. No significant differences were detected in allele or genotype distribution of *VEGF -2578C>A* (*P* = 0.91 and *P* = 0.86, respectively), *-460T>C* (*P* = 0.75 and *P* = 0.93, respectively), *+936C>T* (*P* = 0.58 and *P* = 0.81, respectively) and *KDR* -*604C>T* (*P* = 0.18 and *P* = 0.09, respectively), *1192G>A* (*P* = 0.19 and *P* = 0.16, respectively) and *1719T>A* (*P* = 0.31 and *P* = 0.45, respectively) SNPs between tendinopathy cases and controls. However, the multivariate analysis (after adjusted by age, years of practice in volleyball, gender and pain) showed the *KDR 1192G>A* were negatively associated with tendinopathy, pointing to a lower risk in disease development (Table 3).

**Table 3:** Association analyses of the *KDR 1192G>A* polymorphism in tendinopathy cases compared with athletes without disease.

*KDR 1192 G>A*	Controls (n = 91)	Tendinopathy (n = 88)	*P*-value^a^	OR (95% CI)^b^
	N (%)			
GG	59 (64.8)	67 (76.1)		1^c^
GA	30 (33.0)	18 (20.5)	0.02	0.41 (0.19–0.88)
AA	2 (2.2)	3 (3.4)	0.63	1.61 (0.23–11.5)
GA + AA	32 (35.2)	21 (23.9)	0.04	0.47 (0.23–0.98)

OR, odds ratio; CI, confidence interval

^a^Chi-Square Test or Fisher’s exact test

^b^Adjusted by age, years of practice in volleyball, gender and pain

^c^Reference group.

Table 4 shows the distribution of *VEGF* and *KDR* haplotypes among the athletes (S2 Table). A total of eight haplotypes could be inferred for each gene. After adjusted by age, years of practice in volleyball, gender and pain, there was negative risk association for the development of tendinopathy for the *KDR CGA* and *CAT* haplotypes, compared with the reference haplotype *CGT*.

**Table 4:** Haplotype distributions of *VEGF* and *KDR* in volleyball athletes and their association with tendinopathy risk.

Haplotypes	Controls (n = 182)	Tendinopathy (n = 176)	*P*-value^a^	OR (95% CI)^b^
*-2578C>A*, *460T>C* and *936C>T VEGF*	N (%)			
CTC	109 (59.9)	101 (57.4)		1^c^
CTT	1 (0.6)	2 (1.1)	0.93	1.06 (0.28–3.97)
CCC	5 (2.7)	8 (4.5)	0.53	1.49 (0.44–5.07)
CCT	5 (2.7)	6 (3.4)	0.72	1.58 (0.13–19.5)
ACC	42 (23.1)	45 (25.6)	0.62	1.14 (0.67–1.94)
ACT	17 (9.3)	12 (6.8)	0.38	0.68 (0.29–1.61)
ATT	2 (1.1)	1 (0.6)	0.88	0.82 (0.07–10.1)
ATC	1 (0.6)	1 (0.6)	0.66	1.86 (0.11–31.0)
*-604C>T*, *1192G>A* and *1719T>A KDR*	N (%)			
CGT	48 (26.4)	50 (28.4)		1^c^
CGA	31 (17.0)	19 (10.8)	0.49	0.46 (0.21–0.99)
CAT	15 (8.2)	5 (2.9)	0.02	0.23 (0.07–0.76)
CAA	11 (6.1)	15 (8.5)	0.94	1.04 (0.42–2.59)
TGT	57 (31.3)	65 (37.0)	0.63	1.10 (0.64–1.86)
TGA	12 (6.6)	18 (10.2)	0.79	1.13 (0.46–2.78)
TAT	2 (1.1)	2 (1.1)	0.58	0.53 (0.06–5.04)
TCA	6 (3.3)	2 (1.1)	0.18	0.31 (0.06–1.71)

Number (%); OR, odds ratio; CI, confidence interval

^a^Chi-Square Test or Fisher’s exact test

^b^Adjusted by age, years of practice in volleyball, gender and pain

^c^Reference Group

With regards to pain, traumatic lesion and away from training due to injury, *VEGF* and *KDR* polymorphisms were not associated with clinical symptoms complaints (data not shown).

## Discussion

Tendinopathy is a musculoskeletal injury in which intrinsic and extrinsic factors may contribute to the heterogeneous phenotype. Musculoskeletal injury and subsequent recovery seem likely to result from the interaction of environmental stimuli (training or competition related to mechanical load patterns, or surgery/ unloading) and genotype [20]. Events which precede the disclosure of symptomatic injury have not been completely identified, because may be multifactorial. The injury risk factors in high performance volleyball athletes elite level most likely associated with a training volume, jumping load and repetitive movements on the shoulder are significant risk factors for developing tendon injury [21, 22]. Tendinopathy is a common degenerative musculoskeletal disorder and is related to structural tissue changes, such as increased type III collagen content [23], which may adversely influence the mechanical and material properties of the tendon [24]. Recently, genetic variables have been suggested as intrinsic risk factors for tendinopathy in athletes [5, 7, 25, 26]. The genetic study applied to sport may suggest that specific measures targeted at athletes most likely to develop lesions, can be appropriate to reduce the risk of damage by controlling the training load and the exposure frequency in high mechanical stress actions on the joints. Nevertheless, further investigation must be performed to determine the genes and SNPs may be associated with tendinopathy in athletes [5].

There are large evidence of new blood vessel growth in different types of tendinopathy [18, 27]. The new blood vessels formation are mediated by some angiogenic factors and the angiogenesis is important under distinct pathological aspects, because it is accompanied by the proteolysis of extracellular matrix, hence new blood vessels may invade into the tissue [28]. Since angiogenesis normally is poor in adult normal tendon tissue and increase in degenerative tendon disease [13], this process has been viewed as a potential new target to better define the mechanisms that cause the disease. The VEGF and its receptor KDR are significantly highly expressed in degenerative tendon tissue compared to healthy tendon [16, 17]. Several studies have identified vascular hyperplasia in painful tendons and degenerative processes resulting from sports activity [29, 30]. These findings supported the results of clinical studies using color Doppler examination simultaneously with ultrasonography that showed a neovascularization in areas with tendon injury [31, 32]. Normally, avascular tendon tissue becomes hypervascularized during tendinopathy, which supported a key role for VEGF-KDR signals in the pathological angiogenesis in tendinopathy, because VEGF stimulate endothelial cells and vessels to invade hypovascularized tendon areas [12, 13].

Polymorphisms in *VEGF* and *KDR* may alter protein concentrations [14, 15], influence the process of angiogenesis and consequently may contribute to inter-individual variation in the development of tendinopathy in athletes. The inheritable susceptibility to tendinopathy justifies our interest in identifying SNPs in *VEGF* and *KDR* genes that could lead to an increased risk or severity of the development of the disease, in order to provide additional support for treatment planning of athletes and avoid those athletes from getting away from training due to injury. In the present study, we observed negative risk associations with the development of tendinopathy in athletes for the *KDR 1192G>A* SNP. According to, Wang and colleagues, *KDR 1192G>A* SNP influence the efficiency of VEGF binding to KDR. The *KDR 1192G>A* SNP is located in exon 7, which is the key element of KDR binding domain for VEGF [14]. Moreover, *KDR CGA* and *CAT* (*-604C>T*, *1192G>A* and *1719T>A*) haplotypes were negatively associated with tendinopathy, suggesting an effect of decreased susceptibility. The haplotypes are characterized by the presence of the variant alleles of SNP *KDR 1719T>A* and *1192G>A*, respectively. Polymorphisms in *KDR* may alter receptor activity, influence the process of angiogenesis and consequently contribute to inter-individual variation in the development of tendinopathy in athletes.

Concerning the *VEGF* haplotypes, our data suggested no significant effect on the susceptibility of tendinopathy in athletes. Recently, Rahim and colleagues investigated three SNPs in the *VEGF* (rs699947, rs1570360, rs2010963) and two SNPs in the *KDR* (rs1870377, rs2071559) in relation to the risk of Achilles tendinopathy in two population samples drawn independently from South Africa (SA) and the United Kingdom (UK). Only the *VEGF AGG* haplotype was associated with an increased risk of Achilles tendinopathy in the SA and the combined SA+UK group [33].

The increase of neoangiogeneses besides stimulating the repair process may also contribute to thickening and disorganization of the tendon [34]. The VEGF signals enhance the expression of matrix metalloproteinase (MMP) and reduce the tissue inhibitor metalloproteinase leading to a weakening of the normal tendon structure, decrease of the mechanical strain, and consequently the spontaneous rupture [13]. Therefore, in the same way that the VEGF-KDR induces remodeling in tendon disease, this signal transduction may also initiate tissue degradation processes by increasing MMPs. In addition, Baroneza and colleagues described association of MMP-1 polymorphisms with posterior tibial tendinopathy. MMP-1 polymorphisms may alter collagen types I and III of tendon, which contribute with higher risk of tendinopathy development [6].

A strength of our study is that all population recruited (cases and controls) were from a standardized group of elite volleyball athletes from Brazil, equal physical activity, submitted to controlled training conditions, with similar nutrition and environmental conditions. The main limitation of this approach is that 51% of the controls were sub 18 versus 16% in cases, and 47% of the cases were adults versus 18% in controls. Moreover, 44% of the controls had up to 5 years of practice in volleyball. However, our data can be used to build a database, which can then be used in future investigations to better understand the genetic and extrinsic factors affecting the susceptibility of development of tendinopathy in athletes, and can contribute to identify novel therapeutic targets or personalized training programs to avoid development tendinopathy in athletes. Due to the extensive admixture of the present-day Brazilian population, extrapolation of clinical genetics data derived from well-defined ethnic groups is clearly not applicable to the Brazilians [35].

Training procedures are used in the assessment of athletic performance and injury risk. Thus, practitioners can prepare devise performance enhancement and injury prevention strategies based on association of polymorphisms with tendinopathy by reducing the training volume for those players that may have possibility to express tendinopathy. To knowledge, the *KDR* SNPs may help to develop models that can be used to identify athletes risk for tendon injury.

## Conclusion

In conclusion, our findings provide evidence that the *KDR* SNPs were associated with development of tendinopathy in Brazilian volleyball athletes.

## Supporting Information

S1 Table Overall data of study population. Demographic and clinical variables, and genotypes of *VEGF* and *KDR* in tendinopathy cases and controls.
(XLSX)

S2 Table Haplotypes of *VEGF* and *KDR* in study population. (XLSX)
